# From Invention to Innovation: Risk Analysis to Integrate One Health Technology in the Dairy Farm

**DOI:** 10.3389/fpubh.2017.00302

**Published:** 2017-11-23

**Authors:** Andrea Lombardo, Carlo Boselli, Simonetta Amatiste, Simone Ninci, Chiara Frazzoli, Roberto Dragone, Alberto De Rossi, Gerardo Grasso, Alberto Mantovani, Giovanni Brajon

**Affiliations:** ^1^Istituto Zooprofilattico Sperimentale del Lazio e della Toscana “M. Aleandri”, Section of Florence, Florence, Italy; ^2^Istituto Zooprofilattico Sperimentale del Lazio e della Toscana “M. Aleandri”, Rome, Italy; ^3^Istituto Superiore di Sanità, Rome, Italy; ^4^Consiglio Nazionale delle Ricerche, Rome, Italy; ^5^Cooperativa Lattepiù, Rome, Italy

**Keywords:** dairy chain, cow milk, biosensoristic devices, risk management, risk assessment, food safety, environmental health, Hazard Analysis and Critical Control Point

## Abstract

Current Hazard Analysis Critical Control Points (HACCP) approaches mainly fit for food industry, while their application in primary food production is still rudimentary. The European food safety framework calls for science-based support to the primary producers’ mandate for legal, scientific, and ethical responsibility in food supply. The multidisciplinary and interdisciplinary project ALERT pivots on the development of the technological invention (BEST platform) and application of its measurable (bio)markers—as well as scientific advances in risk analysis—at strategic points of the milk chain for time and cost-effective early identification of unwanted and/or unexpected events of both microbiological and toxicological nature. Health-oriented innovation is complex and subject to multiple variables. Through field activities in a dairy farm in central Italy, we explored individual components of the dairy farm system to overcome concrete challenges for the application of translational science in real life and (veterinary) public health. Based on an HACCP-like approach in animal production, the farm characterization focused on points of particular attention (POPAs) and critical control points to draw a farm management decision tree under the One Health view (environment, animal health, food safety). The analysis was based on the integrated use of checklists (environment; agricultural and zootechnical practices; animal health and welfare) and laboratory analyses of well water, feed and silage, individual fecal samples, and bulk milk. The understanding of complex systems is a condition to accomplish true innovation through new technologies. BEST is a detection and monitoring system in support of production security, quality and safety: a grid of its (bio)markers can find direct application in critical points for early identification of potential hazards or anomalies. The HACCP-like self-monitoring in primary production is feasible, as well as the biomonitoring of live food producing animals as sentinel population for One Health.

## Primary Producers’ Mandate for Legal, Scientific, and Ethical Responsibility in the European Food Safety Frame: The Role of Risk Analysis and Scientific Research

The European White Book for food safety points out the ethic, scientific, and legal responsibility of all food operators, including food primary producers, in guaranteeing the safety of their products. Food safety and traceability have to be ensured at every stage of the food chain, and the primary production is the first critical step (1). In fact, several main food safety alarms in past decades and years (e.g., BSE, VTEC, dioxin contamination of animal feedstuffs) took place in the primary production sector; in the meanwhile, the understanding of the web of interactions among humans, animals, and the environment (One Health) determines the increasing importance of prevention and safety in the primary livestock production.

Dairy farming is among the most complex, and potentially vulnerable, components of farm animal production: the maintenance of good qualitative standards of milk and dairy products still represents a challenge for farmers and manufacturers who, in their turn, ask the scientific community to furnish them with proper tools for hazard identification and risk management. The best strategy to ensure safety calls for implementing preventive approaches, such as good breeding and manufacturing practices or the application of procedures based on the Hazard Analysis and Critical Control Point (HACCP).

HACCP was firstly used in food production in the 1970s, providing precise process control measures for each step of the entire food manufacturing process. The Codex Alimentarius Commission has recognized HACCP as an effective tool to improve safety standards; HACCP identifies priority hazards and allows establishing targeted control systems, thus putting focus mainly on preventive measures rather than on end-product testing (2). HACCP is a food safety system, and ISO 22000:2005 is a food safety management system standard. As described in the Codex Alimentarius, ISO 22000:2005 mainly fits postprimary production/transformation (pasteurization/microfiltration and cheese factories) and, more than HACCP, focuses on policy, standards, targets, communication, and planning.

The application of HACCP-like systems to animal health and primary production still represent the best approach (3). The European Union forced the implementation of HACCP after the revision of the hygiene directives (4–6) and the general food law (7). Currently, HACCP focuses on microbiological hazards and risks, as can be found in public and animal health state instituted plans. HACCP should focus also on hazards of different nature, such as chemical and physical contamination of products and even on animal welfare disorders.

Currently, the European Union recommends primary producers, such as dairy farmers, to apply a HACCP-like program to prevent milk-borne zoonoses; noticeably, the modern concept of zoonoses does include toxicological risks carried over in foods of animal origin (8). However, the application of such programs on dairy farms is still not developed: indeed, implementing new strategies and technologies for the application of HACCP in primary production represents a point of utmost importance. ALERT^1^ is a project funded by the Italian Ministry for Economic Development, and based on the BEST technological integrated bioelectronic system and relevant control charting for early intervention on food chain and the environment (9). Along with a new field and self-instructed technology working in the farm environment, ALERT aims at developing and making available to dairy farmers a modernized risk management framework based on scientific evidence and recommendations by international agencies (9).

In this paper, we define the framework for technology transfer. Indeed, true innovation needs translational activities to make inventions (in this case, the BEST system) be sustainably integrated in complex and dynamics real systems. Through field activities in a selected dairy farm in central Italy, we explored individual components of the dairy farm system to define both opportunities and challenges of the BEST technology transfer. The farm-specific scenario is then considered at a broader spatial scale, together with neighboring farms, in order to highlight possible significant aspects associated to managerial or environmental factors.

The multidisciplinary and interdisciplinary One Health profile (environment, animal health, food safety) of the ALERT project is further amplified by the involvements of technological innovation. Farm characterization and risk analysis are basic inputs to establish a targeted grid of probes of the BEST platform in order to monitor a farm-tailored panel of analytical parameters. Indeed, as all health-oriented innovation initiatives the ALERT framework is complex and subject to multiple variables (10).

## Materials and Methods

The dairy farm (Lazio region, 41°54′47.94′′N, 12°15′48 ′25′′E) object of the present study was selected as representative of a well-conducted, relatively large-sized dairy farm of Central Italy.

The characterization of the farm both as an environment, an animal rearing facility and a segment of the food chain was carried out following the seven HACCP principles (11) during 12 onsite monthly visits to the selected farm, from January to December 2012.

The farm characterization made avail of the checklists elaborated by the Agricultural Agency of the Tuscany region and by National and European Authorities [(12), Welfare Quality-Cattle protocol,^2^ (4)] and currently in force in the official control system. The main topics covered the following:

(1) Farm position and territorial analysis of the macro-area around the farm. The dataset comprises farm position and area, geo-climatic factors, possible pollution sources (e.g., waste disposal sites), presence of neighboring protected areas, presence of endangered species, land usage, main crops, agricultural techniques, previous mycotoxins alerts, hydro-geographic network, and presence of farms and/or factories within a 20-km buffer around the farm. In order to identify possible health risks from zootechnical activities within the buffer area, among the 40 small size (<300 heads) dairy farms and 1 larger farm (>500 heads) identified, three farms were selected based on structural homogeneity, productive capacity, and lower distance from the chosen farm. Milk quality analysis data of these three farms from 2010 to 2013 were collected (Source: Istituto Zooprofilattico Sperimentale del Lazio e Toscana (IZSLT) Laboratory Information System) and statistically analyzed (MedCalc version 12© 1993–2012 MedCalc Software bvba).
(2) General farming conditions. The dataset comprises animal identification, number of heads for each category, structures, conditions of animal barns (ventilation, illuminations, etc.), dry period management, biosecurity, and prevention tools.
(3) Agricultural, fertilizing, and weeding practices, with particular attention to main crops, pesticides management including the risk of groundwater pollution.
(4) Animal nutrition, with particular attention to feed quality, safety, and origin.
(5) Animal health and welfare (anti-microbials and anti-parasitic drugs usage and management, udder health).
(6) Milking techniques and milking parlor hygiene.

Critical points were monitored through routine laboratory analysis with instruments and methods currently used by the Official Control System. Routine laboratory analyses were performed at the “Istituto Zooprofilattico Sperimentale del Lazio e Toscana M. Aleandri” laboratories under a total quality assurance system and were certified by the Italian Bureau for Laboratory Accreditation “Accredia,”^3^ Rome, Italy (number of accreditation 0201). Laboratory analysis covered the following: well water (total bacterial count, coliforms and *Escherichia coli*, heavy metals, pesticides), feed and silage (pesticides, heavy metals, mycotoxins) (13), individual fecal samples (parasitological analysis, *Salmonella* spp., *Campylobacter* spp.), and bulk milk (total bacterial count, somatic cell count, fat, protein, lactose, aflatoxin M_1_, antimicrobial residues) (Table 1) for comparison with the site-specific set of (bio)markers in the BEST Platform (Table 2).

**Table 1:** Analysis performed at the identified points of particular attention (POPAs) and critical control points (CCPs).

Element	CCP or POPA	Indicators/analysis performed	Technique	Reference
Water quality (beverage and cleaning)	CCP	Total bacterial count, coliforms, *Escherichia coli*	Cultural	UNI EN ISO 6222:2001UNI EN ISO 9308-1:2014
		Heavy metals (cadmium, lead) and pesticides residues (florasulam, 2,4-dichlorophenoxyacetic acid, mesotrione, terbuthylazine, desethyl-terbuthylazine, and S-metolachlor)	GC MS, ICP MS	Internal certified method (POS CHI 051 INT rev 0 2011, POS CHI 028 INT rev 4, 2013)

Feed and silage quality	CCP	Heavy metals (cadmium, lead), pesticides residues (florasulam, 2,4-dichlorophenoxyacetic acid, mesotrione, terbuthylazine, desethyl-terbuthylazine and S-metolachlor), and mycotoxins residues (aflatoxin B_1_)	GC MS, ICP MS, ELISA	Internal certified methods (POS CHI 051 INT rev 0 2011, POS CHI 028 INT rev 4, 2013, POS 037 INT rev 0, 2009)

Animal health/zoonoses	POPA	Gastrointestinal pathogens (*Salmonella, Campylobacter, E. coli, Cryptosporidium*)	Cultural, microscopic analysis	OIE Manual for terrestrial animals 2010 cap 2.9

Bulk milk quality	CCP	Total bacterial count, somatic cell count Fat, protein and lactose content Mycotoxins (aflatoxin M_1_) Antimicrobials residues (lincomycin, spectinomycin, marbofloxacin, ciprofloxacin, amoxicillin, flunixin, and 5-hydroxy flunixin)	Opto-fluorometric ELISA Microbiological	Internal certified methods (POS CIP 021 INT rev5 2015). AFNOR DSM 28/02–02/12 Delvotest^®^ and internal certified methods (POS CIP 018 INT rev11 2015, POS CHI 038 INT rev5 2015)

**Table 2:** Set of (bio)markers selected for the site-specific BEST platform.

Non-targeted (indicators of safety/quality)	Targeted (specific analytes, including milk components and residues/contaminants)
Temperature	Calcium ions
pH	Sodium ions
Redox potential	Potassium ions
**Total milk quantity/milk flow**	Iodide ions
Conductivity	Fluoride ions
Aerobic cellular respiration^a^	Chloride ions
Oxygen	Nitrate ions
Carbon dioxide	Ammonium ions
Chlorophyll a fluorescence^b^	**Heavy metals**
Tyrosinase^c^	**Antibiotic residues**
Laccase^d^	**Fat**
**Urease**	**Protein**
Lactate dehydrogenase	**Lactose**
Glucose oxidase	Blood
	**Somatic cell count**
	**Total bacterial count/mastitis-causing bacteria (*Streptococcus uberis* and *Escherichia coli*)**
	**Pesticides**
	**Aflatoxin M_1_**

*Parameters in bold were monitored through laboratory analysis in this study*.

*^a^General toxicity/wholesomeness*.

*^b^Exposure to pesticides inhibitor of photosystem-II complex, including phenyl-carbamate, pyridazinone, triazine, uracils, ureas, benzothiadiazinones, and phenyl-pyridazines pesticide*.

*^c^Exposure to phenolic, organophosphate, and carbamate pesticides*.

*^d^Exposure to phenolic and carbamate pesticides*.

Farm owners and farmers have been formally enrolled in the Consortium of the project ALERT and thus they consented to the collection and use of data. According to EC (14) of the European Parliament and of The Council of 22 September 2010 on the protection of animal used for scientific purposes and the Italian law “Decreto Legislativo 26/2016,” (15) the authors can assert that all the animals involved in the study were exclusively submitted to practices respecting animal welfare and undertaken for the purposes of recognized animal husbandry, in accordance with good veterinary practice. Thus, the study does not require any further specification regarding ethics approval by authors.

## Results

### Farm Characterization: Checklists

#### Farm Position and Territorial Analysis of the Macroarea around the Farm

The selected farm is located in Central Italy and rears high-production Italian Holstein cows (average 9.5 tons milk/cow/year). The farm covers an area of over 350 ha of cultivated land, ranging from a 400 to 500 m high hilly zone to the plain along the Tyrrenian coast. According to Mayr-Pavari definition for phytoclimatic zones, the area lays in the Lauretum zone (warmer subzone, with summer drought). The broader area including the dairy farm is involved in agricultural production (grasslands, woods, cereals and herbaceous crops, olive groves and vineyards).

Concerning nitrogen pollution, the farm lays in a nitrate non-prone zone (Council Directive 75/440/EEC and Italian National Regulations: D.lgs. 152/99 and D.lgs. 258/2000) (16). This area does not include any chemical factories or other potential sources of water and environmental pollution. The analysis highlights the presence of simple cropping systems (dry and irrigated), permanent herbaceous crops (lawns, meadows pastures, and alfalfa *Medicago sativa*), and uncultivated areas with natural vegetation (wild trees and shrubs, and uncultivated fields). The quality of crop does not require specialized use of chemicals in their growing cycle. The absence of specialized fruit orchards, vineyards, and vegetable crops reduces the possible direct contamination by agrochemicals (fungicides, insecticides, herbicides, etc.).

Mycotoxins contamination is considered the most important toxicological risk of the macroarea; nevertheless, contamination of milk can be considered a rare event. From 2009 to 2013, aggregated data of Official Controls for Aflatoxin M1 in bovine raw milk in Tuscany and Lazio Regions reveals 324 (3.3%) samples above the legal thresholds on 9,723 total analyzed samples, with a peak prevalence (9.6%) in September; data about the occurrence of Aflatoxin B1 in feed and silage in the same years showed a prevalence of 100 (12.7%) samples above the legal thresholds out of total 570 analyzed samples (Source: IZSLT Laboratory Information System). These prevalence rates of Aflatoxin B1 contamination events could be overrated by the introduction of feed from other parts of Italy or from abroad.

Milk quality analysis data from 2010 to 2013 are shown in Tables 3–6. Data show a good health status and a substantial similarity among the three farms.

**Table 3:** Average values of fat and protein content, total bacterial count, and somatic cells of bulk milk of the three nearer farms from 2010 to 2013.

Farms	Fat (%)	Protein (%)	Total bacterial count (CFU*1,000/mL)	Somatic cell count (cells*1,000/mL)
1	3.68	3.27	14	239
2	3.79	3.36	53	289
3	3.77	3.40	28	285

**Table 4:** Antimicrobials residues and aflatoxin M_1_ of the three nearer farms from 2010 to 2013.

Farms	Antimicrobials residues (positive samples)	Aflatoxin M_1_ (ng/kg)
1	0	<30
2	0	<30
3	0	<30

**Table 5:** Average values of fat and protein content, total bacterial count, and somatic cells of bulk milk of the three nearer farms per year.

Year	Fat (%)	Protein (%)	Total bacterial count (CFU*1,000/mL)	Somatic cell count (cells*1,000/mL)
2010	3.69	3.32	35	292
2011	3.76	3.35	32	273
2012	3.77	3.37	35	240
2013	3.81	3.36	20	294

**Table 6:** Antimicrobials residues and aflatoxin M_1_ of the three nearer farms per year.

Year	Antimicrobials residues (positive samples)	Aflatoxin M_1_ (ng/kg)
2010	0	<30
2011	0	<30
2012	0	<30
2013	0	<30

#### General Farming Conditions and Animal Housing

The farm is registered due to EC Regulation 852/2004 and authorized for the production of high-quality milk due to the Italian law DM 185/91. The whole milk produced is destined to pasteurization and direct consumption, without transformation. The farm owns 420 total heads (160 lactating cows, 30 primiparous). The animals are correctly identified due to EC Regulation 1760/2001. The farm is composed by six different areas for animal housing: (1) Lactating Cows, (2) Dry Cows, (3) Heifers, (4) Calves (paddock and individual cages), (5) Infirmary, and (6) Grazing land. All the animals (except for calves up to 40 days reared in single boxes) are reared in multiple boxes with an indoor section with permanent hay litter (density 6.5 mq/head) and an outdoor paddock. Bedding is renewed daily (5–6 kg hay/head in autumn and winter and 2–3 kg hay/head in spring and summer) and the hygienic condition is very good. Ventilation and illumination are natural; air flowing is guaranteed by mean of large windows and there is no fecal or ammonia smell in the animal premises.

#### Agricultural Management

The total agricultural area is about 360 ha, while the utilized agricultural area (UAA) is about 350 ha. Such area is involved in the phytosanitary measures that the Lazio Region has issued for the control of the Western corn rootworm (*Diabrotica virgifera virgifera*).

The currently employed crops are listed in Table 7. The final use of the crops is entirely dedicated to animal supply. The main cultivation operations such as tillage, seeding, fertilizing, weeding, herbicide and pesticide treatments, irrigation, hay, and silage are performed without external intervention.

**Table 7:** Main culture and crop production.

Crop	UAA (ha)	Production
Corn	55	Silage
Grass (wheat, barley, triticale)	35	Silage
Alfalfa	55	Silage, hay
Grass (oats, Lolium, clover)	165	Hay
Wheat	40	Grain

Fertilization is performed either with farm’s manure and synthetic fertilizers, such as ammonium nitrate (NH_4_NO_3_) and urea [CO(NH_2_)_2_]. Herbicides and pesticides treatments are carried out with specific products [mesotrion 3.39% (37.5 g/L), S-metolachlor 28.23% (312.5 g/L), terbutilazine 16.94% (187.5 g/L), and florasulam (6.25 g/L)]. Even though treatments are carried out respecting the relevant legal limits, there is the need to monitor the possible pollution of groundwater or crops by the parent molecules or their main by-products, considering also the possible accumulation and mixture effect.

#### Animal Nutrition

100% of forage and silage are produced within the farm, while a varying proportion of grain, protein nucleus, and flour (corn, barley, faba beans, and wheat bran) are purchased outside. There are no different feeding groups for the different production levels; feedstuffs are administered twice a day as unifeed. The unifeed present in the manger is in good condition and particle size is homogeneous. Dry cows are fed only with hay herbage and mineral supplement. The mangers are clean and dry and feed residues are modest. The documents relating to purchased feed and the records of loading and unloading are properly managed and are analyzed once a year. The core and flour are guaranteed as genetically modified organism and aflatoxin-free by the manufacturer.

#### Animal Welfare and Health Management

The farm is officially free from tuberculosis, brucellosis, and enzootic bovine leukosis. Vaccination against clostridial infections is regularly practiced. The parasitic load is evaluated yearly by coprological evaluation, and on rare occasions ivermectin treatments are required. The main health problems are represented by (i) placental retention (8%), (ii) mastitis (5–6%) caused by *Streptococcus uberis* and *E. coli* (17), (iii) lameness and claw disorders (5%), (iv) cutaneous papillomatosis (1%), and (v) neonatal diarrhea reported as a very rare event.

The most used veterinary drugs in the farm are antimicrobials: lincomycin and spectinomycin, marbofloxacin, flunixin meglumine, and amoxicillin. Treated animals are identified on the mantle to ensure the isolation of milk at milking time. The farm is not authorized to hold stocks of drugs; veterinary prescriptions are properly recorded. Nutritional, health, and hygienic status has been assessed for all dry cows, about 10% of lactating cows and 10% of heifers.

#### Milking Techniques and Hygiene

Cows are milked immediately after calving and from 1 week after calving milk is collected in a buklet (during the first week colostrums is collected separately) up to 305 days. Cows are dried through drastic reduction of the feed (straw, hay, little, herbage, and water only) and use of intramammary antibiotics; milking is interrupted abruptly. The whole farm produces an average of 30–35 L/head/day, for a total of 8.5–9.0 tons/head/year. Cows are milked twice a day by two operators.

The parlor consists of two herringbone lines, originally 5 + 5, then extended to 7 + 7, with Afimilk^®^ automatic milking machine adopted in the frame of the ALERT activities and integrated in the BEST platform (42-kPa vacuum level, 60 cycle per minute, pulsation ratio 1:1) with electronic recognition of cows through the use of pedometers. The whole milking process lasts about 3 h (mean time of attack-detachment for each cow is 7–8 min). The operators do not wear gloves during milking and pre-milking teat dipping is not performed. There is no use of oxytocin, even in primiparous cows.

##### Pre-Milking Routine

Udder is washed with drinking water (from municipal aqueduct) and disinfected with chlorhexidine and finally dried with disposable paper. The first streams of milk are usually discarded.

##### Mechanical Milking

Operators attach the milking clusters ensuring a well-balanced contact with teats. Milk is firstly collected in a small collector tank, filling and emptying every 20 s, which conveys the milk into the main cooling tank.

##### Post-Milking Routine

In order to remove/reduce the risk of cross-contamination with contagious mastitis pathogens, a post-milking teat dipping is performed using a filming iodophor disinfectant (IODO PVP FILM) as a barrier preventing bacteria from colonizing teat’s surface and orifice.

##### Milking Machine and Tank Disinfection

Disinfection of the milking machine is performed with an acid–alkaline treatment after each milking. Collection time, temperature, and quantity of the milk are properly recorded.

### Farm Characterization: Flow Diagrams and CCPs and POPAs

The flow diagrams of the production process were drawn. Based on the flow diagrams, critical steps and risk factors for risk management in the farm were identified based on risk assessment.

**Figure 1:**
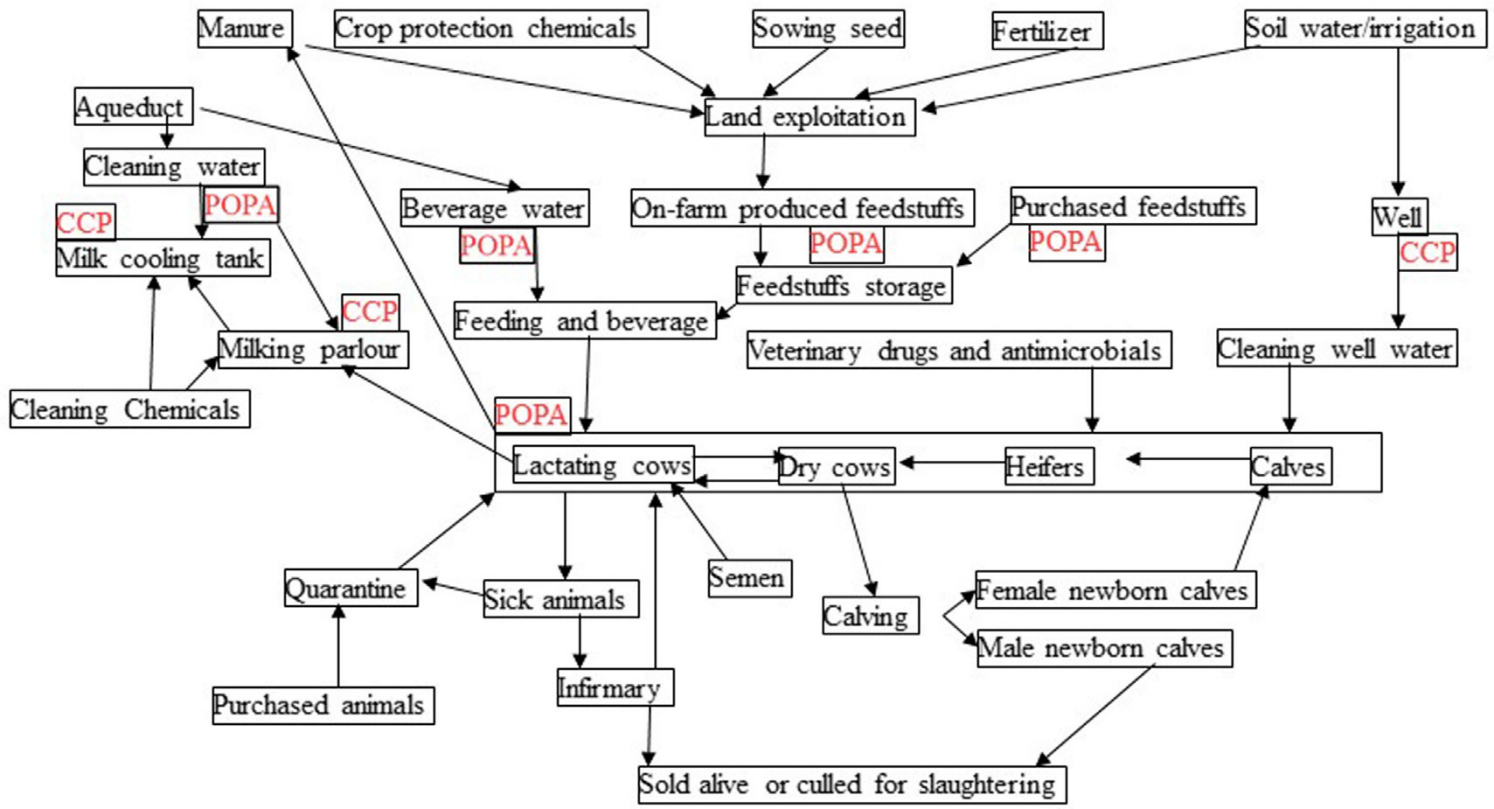
General flow diagram of the production process in the dairy farm in central Italy.

**Figure 2:**
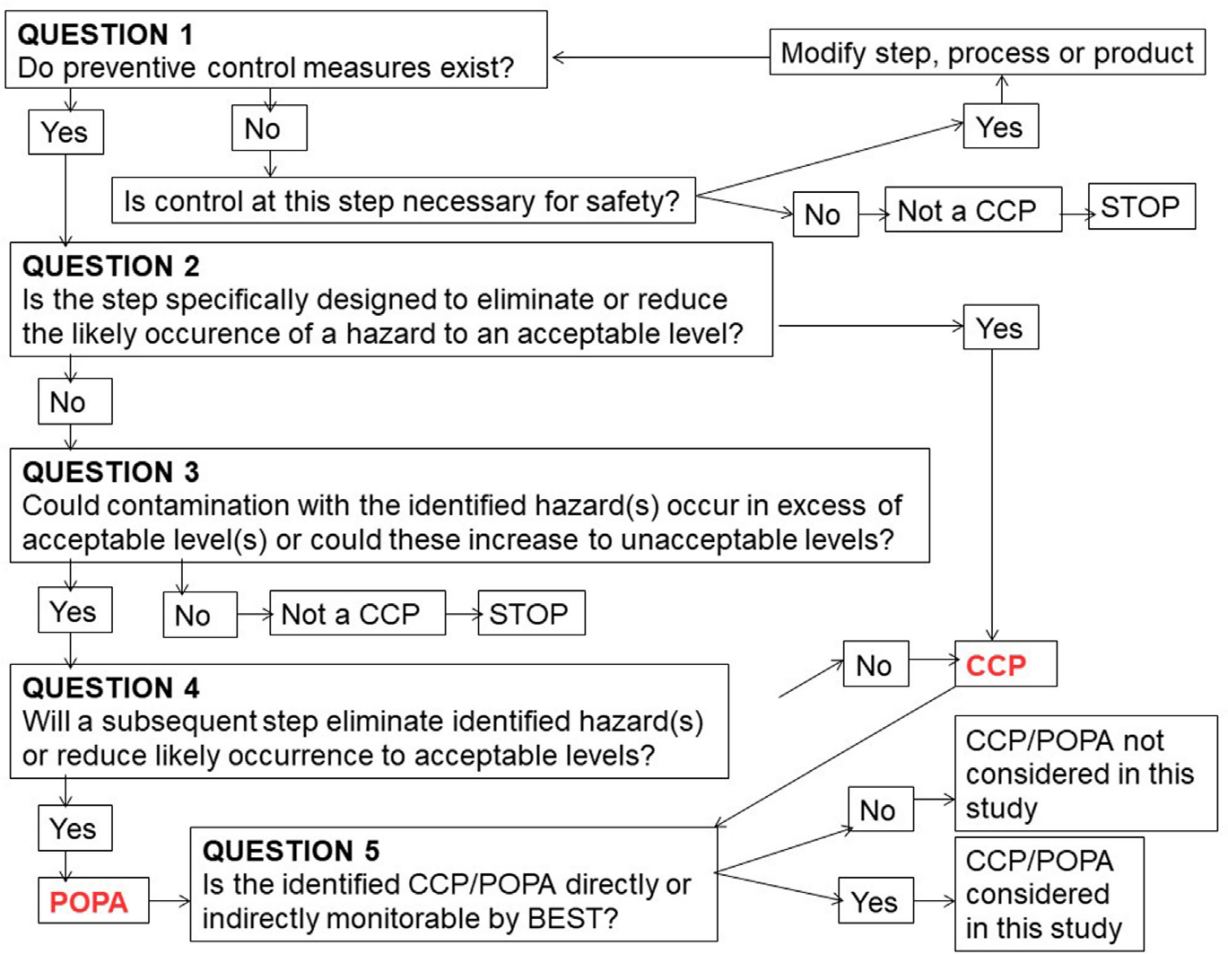
Decision-tree approach in the dairy farm in central Italy. The approach is to determine whether a control point is critical (CCP) or not points of particular attention (POPA). Only POPAs and CCPs monitorable by BEST were considered and monitored.

Critical points associated with a potentially occurring hazard impacting on production were identified and classified as control points [critical control points (CCPs)] or points of particular attention (POPAs) (Figures 1 and 2). In particular, according to the principles and methodology of Noordhuizen et al. (3), CCPs are measurable or observable and have standard external values possibly subject to official regulations (e.g., governing production stoppage) as well as available corrective actions to restore control.

According to the design of the BEST platform, POPAs are critical points where anomalous trends are measurable and, through anomalous variations in relevant control charting, can drive early risk management procedures in HACCP-like plans (9).

Based on the model presented in Noordhuizen et al. (3), the farm management *Decision tree* is drawn, under the One Health view (environment, animal health, food safety), with special attention to POPAs and CCPs that can be monitored with the BEST platform.

### Farm Characterization: Laboratory Analysis at POPAs and CCPs

#### Well Water, Feed, and Silage

Metals, pesticides, and mycotoxins in feed and well water resulted below the respective legal thresholds or below the limits of quantification/detection, except for pirimiphos-methyl—an organophosphorus pesticide found in one feed sample (0.2 mg/kg). Water (both for drinking or cleaning) showed good microbiological standards (18, 19) (Table 8).

**Table 8:** Water quality parameters (mean values).

	Cleaning water	Water at watering^a^
Fecal coliforms	0 MPN/100 mL	0 MPN/100 mL
Total coliforms	0 MPN/100 mL	1 MPN/100 mL
*Escherichia coli*	0 MPN/100 mL	0 MPN/100 mL
Total bacterial count (22°C)	<1 CFU/mL	23 CFU/mL
Total bacterial count (37°C)	<1 CFU/mL	<1 CFU/mL
Fecal streptococci	0 MPN/100 mL	1 MPN/100 mL

*^a^Water collected from drinking troughs*.

#### Coprological Analysis

Zoonotic agents were not detected from any fecal sample.

#### Bulk Milk

In accordance with EC Regulation 853/04, 37 bulk milk samples were processed for total bacterial count (CFU*1,000/mL), somatic cell count (cell*1,000/mL), fat (%), protein (%), lactose (%), aflatoxin M1 (μg/kg), and antimicrobial residues. Data show a good milk quality (20, 21) (Table 9).

**Table 9:** Bulk milk quality.

	Fat (%)	Protein (%)	Lactose (%)	Somatic cell count (cell*1,000/mL)	Total bacterial count (CFU*1,000/mL)	Aflatoxin M_1_ (ng/kg)	Antimicrobials residues
**Mean**	**3.81**	**3.33**	**4.77**	**220**	**38**	<30	**<MRLs**
**SD**	**0.13**	**0.11**	**0.04**	**44**	**20**	–	**<MRLs**
Min	3.51	3.14	4.68	133	12	<30	**<MRLs**
Max	4.07	3.50	4.84	328	101	<30	**<MRLs**

## Discussion

The One Health concept applied to toxicant-related zoonoses requires the analysis of risks in the web of interactions at the environment–animal–human interfaces (8).

No environmental pollution sources were identified by the checklists. The farm is located in a not nitrate-prone area that is suited to agricultural activity, and near to protected natural areas (22). In the surroundings, there are no chemical industries or waste disposal sites, but only small-size dairy farms, characterized by good management and good milk quality standards. Cropping systems do not require a broad use of agrochemicals, making it unlikely a significant contamination of the vegetables used for feeds and of the water system. Groundwater contamination (Table 8) was highly variable and the results may not be representative of any temporal problems, thus highlighting the importance of a in continuum monitoring offered by the BEST.

Overall, the study farm presented a good standard of farming (23), agricultural, and sanitary practices. These observations were confirmed by the results of laboratory analyses. For instance, the absence of residual inhibiting substances and aflatoxin M1 indicate good animal husbandry, good management of feed as well as a conscious use of antimicrobial drugs (24). Aflatoxin alerts have become relatively common in Northern Italy due to climate changes, land usage and cropping errors, inadequate irrigation, parasites and insect attacks, and harvest preservation disorders (25). All these factors may lead to fungal colonization and toxins production. Prevalence may reach peaks higher than 10% of total processed samples. Based on the overall scenario, risk of aflatoxin B1 contamination can be considered mainly during and shortly after summer drought. As the legal thresholds are exceeded, milk have to be destroyed by local Authorities, thus causing important economic losses for farmers.

Breeding techniques ensure good standards of welfare and animal health. Paratuberculosis is widely diffused in Italy; the farm prevalence can be considered quite low, thus highlighting the possible eradication by mean of the new regional prophylaxis program.

Based on the HACCP-like approach and farm management decision tree, the analysis carried out in the sequential POPAs of the farm identified a limited set of farm-specific CCPs. In particular, we consider the following concepts.

(1) Well water should be periodically checked for pollution by synthetic fertilizers (ammonium nitrate and urea), as well as for bacterial contamination; indeed, the management of litter could lead to the risk of fecalization of the groundwater, as suggested by previous finding of “environmental” bacteria in fore-milk and water (*E. coli*). Well water is vulnerable to pollution by pesticides and their degradation products; even though the analyses did not reveal the presence of residues, monitoring is warranted.
(2) Bulk milk represents the end-stage product of dairy farms. Information gathered on bulk milk is obviously pivotal for food safety (e.g., residues, contaminants, somatic cells, and total bacterial count). Finally, milk may represent an indicator of the environmental quality, both of surrounding areas out of the farm (e.g., residues of heavy metals or pesticides) and inside the farm as determined by farming management systems (e.g., residues of veterinary drugs, disinfectants, aflatoxin M1). Overall, milk can be considered as a real “One Health” biomarker as it can provide a cluster of data relevant to food safety, animal health, farming management and environmental quality (26), thus protecting health and preventing food losses.

Databases of laboratory analysis provide interesting information for investigation and comparison in other farm systems.

The BEST system of early (bio)markers of anomalies can be applied as monitoring system at well water (POPA) and bulk milk (CCPs). The grid of markers (in environmental matrices) and biomarkers (in animal fluids) of the BEST platform (sensors and biosensors) is flexible, so as to host new probes depending on site-specific requirements (27–30). The grids of (bio)markers recommended in the selected POPAs and CCPs of the study farm are reported in bold in Table 2. Indeed, through new (automated) technologies like BEST account for the potential for “cocktail” effects from multiple residues and contaminants with different half-lives, metabolism, persistence, tissue accumulation, and targets. Multiarray signals covering oxidative stress, mitochondrial dysfunction, interactions with nutrients (vitamins, essential elements) leading to lipid/glucose dysmetabolism are promising sets of biomarkers early alerting on significant anomalies occurring in the farm, with important One Health implications.

The use of BEST at watering, milking parlor, and bulk milk is expected to facilitate daily monitoring of farm environment and management, milking efficacy and efficiency, process hygiene, and milk safety. Indeed, the user-friendly and self-instructed (by control charting) BEST system operating on-line and providing timely and continuous information can support the maintenance of production quality (31) as well provide early warnings that trigger appropriate decision trees (32).

Daily maintenance of a good farm management means time and cost-effective preparedness to unwanted and/or unexpected events of both microbiological and toxicological nature. Prevention strategies based on an HACCP-like self-monitoring systems empowering primary food producers (33) and providing measurable (bio)markers to monitor anomalies (including toxicological hazards) in critical points are crucial for translational science in real life. Scientific advances in risk analysis-driven biomonitoring of sentinel animals (26) are exempla of health-oriented innovation in primary production that exploit the “One Health” framework (10).

## Conclusion

The application of risk assessment using POPAs and CCPs for farm management is a valuable initiative to overcome challenges of translational science in (veterinary) public health. The understanding of complex systems is a condition to accomplish true innovation through new technologies. In the case of One Health technology, biomonitoring of sentinel animals like food producing animals is crucial. The framework discussed in this work demonstrates how the development of an HACCP-like self-monitoring system based on measurable markers in critical points of the primary production chain and in live animals is feasible. Scientific advances in risk analysis can be applied to prevent toxicant-related zoonoses in daily primary production of food, with simultaneous benefit (One Health) for the protection of human, animal, and environmental health.

## Ethics Statement

Farm owners and farmers have been formally enrolled in the Consortium of the project ALERT and thus they consented to the collection and use of data. According to EU Directive 2010/63 of the European Parliament and of The Council of 22 September 2010 on the protection of animal used for scientific purposes and the Italian law “Decreto Legislativo 26/2016,” the authors can assert that all the animals involved in the study were exclusively submitted to practices respecting animal welfare and undertaken for the purposes of recognized animal husbandry, in accordance with good veterinary practice. Thus, the study does not require any further specification regarding ethics approval by authors.

## Author Contributions

All the authors substantially contributed to: (1) the conception of the work and the acquisition, analysis of interpretation of data; (2) drafting and revising critically the work for important intellectual content, (3) final approval of the version to be published; and (4) agreement on accountability in all aspects of the work, in ensuring that questions related to the accuracy or integrity of any part of the work are appropriately investigated and resolved.

## Conflict of Interest Statement

The authors declare that the research was conducted in the absence of any commercial or financial relationship that could be construed as a potential conflict of interest.

## Footnotes

^1^http://www.alert2015.it.

^2^http://www.welfarequality.net.

^3^http://www.accredia.it/accredia_labsearch.jsp?ID_LINK=293&area=7&dipartimento=L%2CS&.
